# MSFP: undergraduate ‘collaborate-from-home’ research in macromolecular structure and function

**DOI:** 10.1093/bioadv/vbad074

**Published:** 2023-07-05

**Authors:** Constance J Jeffery

**Affiliations:** Department of Biological Sciences, University of Illinois at Chicago, Chicago, IL 60607, USA

## Abstract

**Summary:**

When the COVID-19 crisis shut down most undergraduate research opportunities, the Macromolecular Structure and Function Research Experiences for Undergraduates Program provided a mentored research experience on the topic of Macromolecular Structure and Function and training in professional skills to assist the participants in pursuing a degree and a future career in STEM. The fully online, remote, computer-based program was funded by the USA National Science Foundation. It involved faculty at four geographically distributed institutions specializing in diverse but complementary approaches to study macromolecular structure and function. Importantly, its online ‘collaborate-from-home’ format made it accessible to students during the pandemic to participate fully in the research, professional development and other activities of the program. This project can also serve as an example for future remote, online projects that would especially be helpful for students who do not have access to similar programs at their universities, cannot travel to attend a summer program, have physical challenges that make it difficult for them to work in a lab or students whose research opportunities are limited due to the war in Ukraine. The lessons learned with the Macromolecular Structure and Function REU program can provide helpful information for ISCB members to set up similar programs to serve additional students.

**Availability and implementation:**

More information and resources are available on the project web site http://jefferylab.moonlightingproteins.org.

**Supplementary information:**

Supplementary data are available at *Bioinformatics Advances* online.

## 1 Introduction

The COVID-19 crisis eliminated most opportunities for undergraduate research experiences for more than a year. Undergraduate research opportunities are critical for keeping students in the STEM degree and career pipeline. In the summer of 2021, funding was received from the USA National Science Foundation for a Research Experiences for Undergraduates (REU) Macromolecular Structure and Function Project (MSFP) for seven students. The online, remote, computer-based program provided high quality mentored research experiences and training in professional skills to assist the participants in pursuing a future career in STEM at a time when many other opportunities had been lost due to the pandemic.

The intellectual focus was Macromolecular Structure and Function. In recent years, there has been a dramatic expansion in the number of known macromolecular sequences and structures. The ability to make full use of this information has been hindered by the fact that biochemical or biophysical characterization is feasible with only a small percent of them, but advances in computer-based algorithms and analytical methods provide complementary methods that greatly expand our ability to learn what macromolecules look like, what they do, how they perform their functions and how they are regulated. Computational methods can also be used to elucidate molecular mechanisms that are beyond the resolution of experimental techniques.

## 2 Organization plan

The program was conducted online with an interdisciplinary team of research mentors who are located at four universities. The mentors are experts in a variety of computer-based methods and provided projects that enabled participants to learn about cutting edge research questions, approaches and methods. Constance Jeffery (University of Illinois at Chicago) was the lead PI and also mentored student participants in research projects. The research in her lab combines X-ray crystallography to solve protein structures with computational methods to identify and compare features related to function in protein sequences and structures, for example catalytic activity or RNA binding. Fatemeh Kashaghi (University of Illinois at Chicago) studies structural and functional properties of ion channels using computer simulations, for example molecular dynamics simulations to study the features involved in selectivity of ions for transport. Kimberly Reynolds (University of Texas Southwestern) uses statistical analysis of amino acid conservation and co-evolution to identify regions most critical to protein function. Milo Lin (University of Texas Southwestern) develops theoretical models and computational approaches to predict the mechanisms of protein folding and aggregation. Esmael Haddadian (University of Chicago) uses molecular dynamics simulations to study the dynamic behavior and aggregation of proteins. Heather Pinkett (Northwestern University) uses X-ray crystallography, computational biology and bioinformatics to study ATP-binding cassette transmembrane transporters. These proteins use ATP hydrolysis to move substrate molecules across cell membranes. Although the student participants worked individually with the research mentors, a common research theme of macromolecular structure and function enabled the participants to be part of a cohort who worked together during the first Welcome Week, during workshops and while preparing oral, written and poster presentations of their research and results.

The program’s leadership team also included a ‘Near-Peer Mentor’, Nicole Curtis, who was a graduate student with research experience in macromolecular structure and function and was a vital part of the program. She met online with the participants and the PI each week in a Group Meeting and had weekly one-on-one online wellness check-In advising sessions to assist student participants in dealing with challenges and answer questions in a less formal way than with the faculty mentors (Linn, 2015; Strawn and Livelybrooks, 2012). She provided guidance in preparation of the elevator pitch, abstract, poster, final paper and oral presentation and assisted with organizing some of the professional development presentations and activities.

The student participants were undergraduates from colleges across the USA, including Puerto Rico, Massachusetts, California, Illinois, New York and Texas. Although the NSF funded REU programs are usually open to undergraduate students of all years, the participants for this special RAPID REU opportunity were students for whom this was their last summer of eligibility to participate in undergraduate research and for whom the pandemic eliminated most other opportunities. NSF-funded REU projects are also strongly encouraged to include students from groups who are under-represented in STEM careers (African Americans, Hispanics, Native Americans/American Indians, Alaska Natives and Native Hawaiians or other Pacific Islanders) and students from institutions where opportunities to participate in undergraduate research are limited.

A short list of potential participants was selected based on their applications, transcripts, letters of reference and essays describing their interest in the research topic as well as the potential impact of this program on their education and career plans. Finalists were then interviewed by the PI to make the final selection. Because it was important that students started with a basic knowledge of macromolecular structure and function before starting this short summer program, students were usually biology or chemistry majors, although some physics and computer science majors would also have sufficient background to participate. An expectation of good grades in courses in biochemistry and/or molecular biology, as well as basic biology, chemistry and physics, also helped in selecting students who would do well in understanding the program’s introductory material as well as master the methods needed to participate successfully in a summer research project.

Information about REUs are usually posted on the NSF ‘Search for an REU’ web site (https://www.nsf.gov/crssprgm/reu/reu_search.jsp). Due to the short lead time for this RAPID REU, the PI mainly recruited students by contacting colleagues at community colleges and primarily undergraduate institutions, including members of the Biophysical Society’s Primarily Undergraduate Institution Network. To help spread the word to students who are members of groups who are under-represented in STEM, she also contacted faculty and staff members involved in several undergraduate initiatives at UIC and asked them to forward information to eligible students at UIC and through their network, for example, the UIC Latin American Recruitment and Educational Services Program, CIM^2^AS! (Cultural Immersion in Monarchs and Milkweeds Advancing Science Education) and the UIC Summer Research Opportunities Program (SROP). In the future, information about REUs, internships and similar opportunities can also be posted for free on the new American Chemical Society Get Experience platform (Getexperience.acs.org).

## 3 Funding of the project

The program received funding as a National Science Foundation (NSF) Research Experiences for Undergraduates (REU) under the Rapid Response Research (RAPID) funding mechanism. The RAPID funding mechanism is used for proposals having a severe urgency or a need for a quick-response and was used because of the need for such online programs to be set up quickly during the pandemic. Following NSF budget requirements, a total of 1 month of summer salary can be provided, so the PI and the near-peer mentor each received 2 weeks of salary. Student participants received a stipend for 8 weeks and a research allowance for computer and/or internet needs. A small amount of funds was also available for publications, poster printing and similar costs.

## 4 Plan for the summer

The program was based on the PI and mentors’ significant experience in mentoring diverse undergraduate students in both online and in-person research projects (Jeffery, 2021; Jeffery and Ozturk, 2021) as well as methods used in a proven cyber-linked geographically distributed research experience (Alford, 2017) and programs successfully converted to virtual in the summer of 2020, including Project SEED (Warner, 2020) and the UIC SROP, in which the PI mentored two students during the first summer of the pandemic.

As part of the preparation, mentor roles and expectations were communicated through an online mentor orientation and a booklet. These included involving students in meaningful ways in research, meeting with them at least twice per week to monitor progress, providing feedback and assistance and helping participants prepare a paper and oral presentation.

To prepare student participants for full participation, the first week of the 8-week program was organized as a Welcome Week that included the MSFP Program Orientation to meet fellow participants and mentors and receive more detailed program information, Group Tutorials/Bootcamp with an Introduction to the Research Experience and background information about macromolecular structure and function through a series of short lectures, discussions, hands-on bioinformatics experiences and a workshop on tips for a successful experience in the program, for example, how to keep a lab notebook (Supplementary Materials—Parts 1 and 4). For example, background information on protein structure included a review of amino acids, protein primary, secondary, tertiary and quaternary structure, hydrogen bonds and other interactions, domains and motifs, binding, enzyme catalysis, inhibition and regulation, transmembrane protein structure and function, the fluid mosaic model of membrane structure and hydropathy plots. Students were given a paper about the protein structure of ACE2 (Towler et al., 2004) and worksheets to fill in to answer questions about the protein structure and function. Another workshop included information about how to use online databases and servers with an activity in using those resources to analyze and answer questions about ACE2 and other example proteins, for example using UniProtKB (The UniProt Consortium, 2017), BLAST (McGinnis and Madden, 2004) for finding homologues, including homologues in the PDB, and creating multisequence alignments, the MoonProt Database (Chen, 2021) and the TMHMM (Krogh, 2001) server for locating predicted transmembrane helices (Supplementary Materials—Part 5). A tutorial on the Protein Data Bank (Berman, 2000) included an exercise in using the annotation to learn about the protein and using the protein structure graphics to answer questions about a protein structure. Background reading included the Structures of Life booklet from NIH (The Structures of Life, 2007). Other topics covered in the orientation included using PubMed to find journal articles, how to keep a lab notebook, how to read a journal article, tips for students to ensure productivity in research and other tips for undergraduate research (Falcinelli, 2015).

During the remaining 7 weeks of the program, student participants came together for two weekly group meetings, workshops and other activities that enabled participants to interact with each other, the PI and the near-peer graduate student mentor. These meetings helped participants remain connected, discussing research, informal exchanges of information, supporting student progress and making sure that students were adjusting well to the program. Weekly Individual Wellness Check-ins with the near-peer mentor helped in coaching the students in planning and completing assignments and identifying concerns and challenges. Workshops provided professional skills training about Reading a Scientific Paper, How to Keep a Lab Notebook, Preparing and Delivering a Seminar, Writing Abstracts and Scientific Papers, Preparing and Presenting Scientific Posters, How to Give an Elevator Pitch, Applying to Graduate School, Science Careers, Ethical Issues and Responsible Conduct of Research, Diversity and Inclusion, Creating and using an Individual Development Plan (FASEB, 2012) and Responsible Conduct of Research. A panel discussion with current graduate students helped participants learn about the graduate school application process, what to expect in graduate school, applying for fellowships, finding the right advisor and strategies for success.

### 4.1 Information about projects

Thanks to the valuable contributions of the faculty mentors, most of the participants’ time was devoted to learning how to do research and gaining experience in research in biochemistry, biophysics and computational biology. Students participated in full-time mentored research projects using state-of-the-art computer-based methods to advance understanding of the structure and function of DNA, proteins and other macromolecules. Examples of projects include making use of recent cryo-electron microscopy data in molecular dynamics simulation to study conformational changes in an enzyme, docking of peptides to a substrate binding protein, use of statistical coupling analysis with other computational and bioinformatics tools to identify the role of protein sectors in conformational dynamics in the mechanisms and evolution of allosteric regulation of ligand binding affinity, use of UniprotKB (The UniProt Consortium, 2017), the Protein Data Bank (Berman, 2000), PyMOL graphics (The PyMOL Molecular Graphics System, Version 2.0, Schrodinger, LLC) and BLAST (McGinnis and Madden, 2004) multisequence alignments to study enzymes that moonlight as RNA binding proteins, use of Consurf (Ashkenazy, 2016; Glaser, 2003) to study conservation of protein sequences and regions of protein structures during evolution, and modeling of the multivalent interactions between proteins involved in liquid–liquid phase separation to understand the characteristics and factors that affect their behavior. Participants’ poster titles illustrate the results from their research projects (Table 1).

**Table 1.** Examples of poster titles for student projects

Jorge Villa, E. Haddadian, “Analysis of the Insulin Degrading Enzyme Conformational Changes Between Closed and Open State; A Molecular Dynamics Study”
Jaynia Garcia, K. Rivera, J. Greenwood, H. Pinkett, “Computational Docking Study of Substrate Binding Protein SapA”
Cesar Siete, N. Curtis, C. Jeffery, “Proximity of RNA Binding Sites with Catalytic Features in Moonlighting Proteins”
Carolina Ortiz, N. Curtis, C. Jeffery, “Structural Features of RNA Binding Sites in Moonlighting Proteins”
Amina Rizwan, J. McCormick, K. Reynolds, “Are Sector-Connected Surface Sites Involved in Allosteric Control of Transport Proteins?”
Andres Lozano, M. Lin, “Characterization of Liquid-Liquid Phase Separation Through SUMO and SIM Protein Interactions”

Student names are underlined.

### 4.2 At the end of the program, a virtual research

*Symposium and Award Ceremony* was held for participants, and the mentors and members of the mentors’ labs were invited. Participants presented their research accomplishments as oral presentations. Participant’s posters were printed and mailed to the students for use in presentations at their home universities and/or scientific meetings. Participants also received printed certificates for participation and a printed abstract booklet.

### 4.3 The program also included project evaluation and Reporting

To check the project was progressing satisfactorily, short questionnaires were filled out by the participants and mentors (Weeks 3, 6 and 8) (Supplementary Materials—Parts 2 and 3). Participants were also encouraged to give feedback at the weekly meetings with the PI and near-peer mentor. Overall assessment of the program used the NSF’s web-based Student Assessment of Their Learning Gains Undergraduate Research Student Self-Assessment (Hunter, 2009; Weston and Laursen, 2015). Students will be tracked after the program in order to determine career paths.

## 5 Educational benefits of the format of the program

Overall, the format used for the program had several benefits. One major advantage over an in-person REU program is that the research topics were not limited to the research of faculty at one university who were willing to mentor a student over the summer months. Instead, students were able to learn directly from faculty who are experts in several cutting-edge bioinformatics topics by including faculty located in geographically diverse universities. In addition to the individual attention with mentors to learn about their research subjects, the student participants also came together as a group to learn about additional topics with their peers each week. During the student meetings, the students could practice oral and written presentations with their peers, and they could practice explaining their subject of their research to someone who is not working on the same project. The arrangement of meeting each week for workshops given by multiple professor and graduate student presenters also made it possible to cover many more topics in both bioinformatics and professional skills development than a student might normally learn about when doing an undergraduate research project in a lab.

The experience and the materials that were prepared for the program can contribute in general to bioinformatics education. The orientation and workshop materials on macromolecular structure and function and professional skills development are available on the project web site (http://jefferylab.moonlightingproteins.org) for use by other professors for developing similar programs. An abstract booklet describing the projects completed by the participants is also posted, and it was suggested that, in future programs, a list of tools and websites that participants use can be added to each of the project abstracts. The program’s online format can also be especially be helpful for bringing bioinformatics educational and research experiences to many more students who might not have access to these kinds of programs at their universities, might not be able to travel to a university for a summer program, have physical challenges that make it difficult for them to work at a lab bench, or students whose research opportunities are limited due to the war in Ukraine (the PI has another ongoing remote project with a student in Ukraine). Several types of projects in the summer program can be modified to provide research experiences and professional development to one student or many students and can be tailored to fit an 8-week summer project or one or more semesters by adjusting the research topics to study one protein or many and using one type of analysis or many. In addition, interdisciplinary projects combining concepts and experiences from bioinformatics, computer science, biology, physics and chemistry in a student-centered welcoming research program can help encourage women and members of other groups that are under-represented in these fields to gain research experience and continue in academics and careers in STEM.

## 6 Experience gained from the program

In the evaluations and other feedback about the program, the student participants and mentors described the experience they gained from participation. Students described learning about scientific projects and how computers are used in biology and gaining a deeper understanding about topics studied in their classes. There was also strong favorable feedback about some of the workshops, especially presentations about graduate school and how to apply to graduate school, the panel discussion with current graduate students and how to give an elevator pitch, create posters and prepare and give talks. After asking students what additional topics they would like to learn about, the organizing team added several workshop topics, for example gap years and how to find a project and funding.

The mentors gained experience in working with undergraduate students from a variety of educational backgrounds and levels of experience, and they identified several potential improvements for future programs. Students were selected who had good grades in courses in biochemistry and molecular biology, as well as basic biology, chemistry and physics courses, but, for some of the research projects, students should also have more experience in writing or at least working with computer code before starting the program. If students have enough background, including younger undergraduates would enable continuation of projects into multiple years of undergraduate study, which could result in more opportunities for publications and presentations. Mentors also identified some topics to expand in the Welcome Week ‘bootcamp’.

This special RAPID REU was funded for only 8 weeks. In the future, the usual 10 weeks REU duration would enable the students to work longer with the mentors to learn more about concepts and methods and complete a larger project. More time would be available for receiving feedback and suggestions about drafts of presentations and posters from peers. In addition, more evaluation of the final projects, presentations and posters by the mentors and peers could provide additional feedback to the participants and also aid in the overall evaluation of the program.

## 7 Challenges in an online program

There can be challenges to an online research and professional development program. Initially, it appeared that the research topics would be limited to computational or theoretical structural biology. While students do not participate in hands-on ‘wet lab’ research, many research areas in biology and chemistry involve a significant amount of computer-based steps. Undergraduates can assist in projects where data were collected from ‘wet lab’ experiments by members of the mentor’s lab, for example solving and refining protein structures using X-ray diffraction data.

Challenges in computing resources and internet bandwidth requirements were met in two ways. The grant funding included a research allowance to cover computer and internet expenses so that participants would have sufficient computing resources and Wi-Fi to interact with mentors and other members of the program and participate in online group meetings. When more advanced computer resources were needed for the research projects, the student participants logged in remotely to resources used by the mentor’s labs, for example the lab’s computers or computer centers at the mentor’s university.

Keeping undergraduate students engaged can also be a challenge for an online program. The use of essays, letters of reference, interviews and transcripts in the application procedures helped identify students who were strongly motivated to participate fully in the program. Combining a research project that involved frequent meetings with the mentors, multiple group meetings with the PI and other participants, and weekly meetings with the near-peer mentor also helped keep the participants involved. The use of a variety of activities, including ice-breakers, during the group meetings also helped encourage participation in online meetings. Deadlines for assignments (abstracts, posters, etc.) and evaluations by both the participants and the mentors were roughly biweekly. If needed in future programs, it might also be possible to have the stipend paid in smaller portions and based on participation and completing specified goals along with a favorable evaluation by the research mentor.

## 8 Outcomes

In addition to providing research experience and professional training for the participants, the program led to additional products. Several research publications are in preparation about projects the students participated in, and some of the students are expected to be authors. The project web site makes some of the orientation and workshop materials and the symposium abstract booklet available for other students interested in macromolecular structure and function or professional skills development (http://jefferylab.moonlightingproteins.org). These materials can also be used by other professors for training members of their labs and for developing similar research and training programs. Each student participant prepared a scientific poster about his/her project that was printed so that they can share them with their home departments and present them at meetings. Posters including results from some of the student participants were also presented by host labs at the 2022 Biophysical Society and Protein Society meetings. Oral presentations resulting from the program included an invited seminar (via zoom) ‘Various Ways to Pursue a Gap Year(s): An Overview’ by Nicole Curtis, the near-peer mentor, for undergraduates at Loyola Marymount University. Jaynia Garcia presented her research at the Southern California Conference for Undergraduate Research. Participation in this REU also helped student participants to prepare for the next stages in their research training and careers: Cesar Siete, Jaynia Garcia and Carolina Ortiz were accepted into Post-Bac programs.

## 9 Summary

This REU program in Macromolecular Structure and Function provided undergraduate students with an online, computer-based experience integrating cutting-edge research and training, mentoring and professional skills development when many other opportunities had been lost due to the pandemic. The student participants helped in the production of new knowledge to advance understanding of macromolecular structure and function, evolution, stability, dynamics and mechanisms of signaling, transport, assembly and regulation. The students, who are from groups who are under-represented in science or from schools with limited research opportunities, learned how research is conducted and gained vital training, mentoring and professional skills development to enable and encourage these talented students’ interest in science and to continue in graduate school or professional school and a future career in science and help prevent the loss of talented students from the STEM pipeline. These experiences with the MSFP REU program can provide helpful information for members of the ISCB to set up similar remote, online projects programs to bring bioinformatics research to many additional students.

## Informed consent

All of the mentors, student participants and the near-peer mentor mentioned in the article have given consent for use of their names.

## Supplementary Material

vbad074_Supplementary_Data

## Data Availability

A checklist of topics and resources used in workshops and tutorials as well as examples of evaluation sheets and workshop and tutorial worksheets are available at http://moonlightingproteins.org and included in the Supplementary Information.
