# Supplementary material for: MSFP: undergraduate ‘collaborate-from-home’ research in macromolecular structure and function
Source: Bioinform Adv. 2023 Jul 5;3(1):vbad074. doi: 10.1093/bioadv/vbad074 (PMC10374487; doi:10.1093/bioadv/vbad074)
Supplement: vbad074_Supplementary_Data [file vbad074_supplementary_data.docx]

**Supplementary Material**

**MSFP: Undergraduate “Collaborate from Home” Research in Macromolecular Structure and Function**

**C. Jeffery**

**Supplementary Materials**

The Supplementary Materials include a checklist of topics and resources used in workshops and tutorials (Part 1). Examples of evaluation sheets and workshop and tutorial worksheets are included (Parts 2 – 5).

**Supplementary Materials – Part 1 - Checklist of topics and resources**

The following outline includes topics covered in Orientations and Workshops along with some tips on activities to include in the workshops. References to some resources and publications about some of the topics are included. Additional information about some topics was through personal communications or was found online in unpublished sources.

**Introduction to Macromolecular Structure and Function**

This included a review of amino acids, protein primary, secondary, tertiary, and quaternary structure, hydrogen bonds and other interactions, domains and motifs, binding, enzyme catalysis, inhibition, and regulation, transmembrane protein structure and function, the fluid mosaic model of membrane structure, and hydropathy plots

Lehninger’s Principles of Biochemistry chapters on:

- Amino acids, peptides, and proteins
- The three-dimensional structure of proteins
- Protein function
- Enzymes
- Nucleotides and nucleic acids
- Biological membranes and transport

The Structures of Life (2007) NIH Publication No. 07-2778. U.S. Department of Health and Human Services. National Institute of General Medical Sciences.

Some faculty mentors gave oral presentations about their research with an introduction to the methods used (for example, molecular dynamics).

**Databases, Servers**

See example worksheet in Supplementary Materials - Part 5

UniProt - The UniProt Consortium (2017) UniProt: the universal protein knowledgebase. Nucleic Acids Res., 45, D158-D169.

BLAST - McGinnis,S. and Madden,T.L. (2004) BLAST: at the core of a powerful and diverse set of sequence analysis tools. Nucleic Acids Res., 32, W20-5.

TMHMM - Krogh,B. Larsson,G., Heijne,G. and Sonnhammer, E.L.L. (2001) Predicting transmembrane protein topology with a hidden Markov model: Application to complete genomes. Journal of Molecular Biology*,* 305, 567-580.

MoonProt - Chen,C., *et al.* (2021) [MoonProt 3.0: an update of the moonlighting proteins database.](https://www.ncbi.nlm.nih.gov/pubmed/33245761/)Nucleic Acids Res., 49, D368-D372.

**Determining macromolecular structure**

X-ray Crystallography

The Structures of Life (2007) NIH Publication No. 07-2778. U.S. Department of Health and Human Services. National Institute of General Medical Sciences.

**Intro to research and tips for a successful research experience**

Falcinelli,S. (2015) 11 Tips for your first undergraduate research experience. ASBMB Today, May 2015, 36-36.

Tips on how to keep a lab notebook – See Supplementary Materials – Part 4

Sample lab notebook page

**How to read a journal article**

Finding journal articles with Pubmed

Purugganan,M., Hewitt,J. How to Read a Scientific Article. Cain Project in Engineering and Professional Communication.

**Presentation about Gap year**

With tips on how to find a gap year research opportunity, how to contact faculty members

Presentation by a graduate student who had worked as a technician during a gap year

**Writing a scientific paper**

Provide students with an example of a scientific manuscript

How to Write Your First Paper (2019) Biophysical Society Newsletter, June 2019, 10-11.

Jenkins,S. (1995) How to write a paper for a scientific journal. Australian Journal of Physiotherapy, 41, 285 - 289

**Writing an abstract**

McKee,K. (2018) How to write a scientific abstract (online, no publication information)

**Workshop on writing and giving a seminar**

Tips for Great Oral Presentations. Biophysical Society Newsletter, November 2019, Page 8. (and references therein)

Westberg,J., Jason,H. (1991) Making Presentations. Boulder, CO: Centre Communications.

**Ethics**

Code of Ethics for Undergraduate Research, Copyright © 2017 The Council on Undergraduate Research.

Carpi,A., Egger,A.E. (2009) Scientific Ethics, Visionlearning Vol. POS-2.

**Graduate school**

Panel discussion with current graduate students

What do students do in graduate school

How to apply to graduate school

How to pay for graduate school

**Diversity**

Kinds of diversity, benefits to science, discrimination, dealing with discrimination, building a network, allyship

**Making and Presenting Posters**

Provided an example of a poster

Vega,Q. (2015) 10 reasons your poster will impress and amaze at the annual meeting. Quinn Vega, ASBMB Today, March.

**Presentation on making elevator pitches**

For examples, see online videos of 2012 ASCB elevator pitch contest winners – Kiani Gardner, Michael McGuire,

**Creating an IDP**

Clifford,P.S., Fuhrmann,C.N., Lindstaedt,B., Hobin,J.A. (2013) An individual development plan will help you get where you want to go. Physiologist, 56, 43-4.

Web site for American Chemical Society IDP:

<https://chemidp.acs.org/assess-yourself>

**Mentor and Student Participant Orientation topics – see Supplementary Materials Parts 2 and 3**

Dates of program activities, program goals, contact information, mentor roles, evaluations, restrictions,

Suggestions for Fostering a Productive Faculty-Student Relationship

Checklist for Students to Ensure Productivity – See Supplementary Materials – Part 4

Potential discussion topics for mentors and mentees (for example, topics in effective use of time, graduate school, professional responsibility, professional development, balancing professional and personal life)

Student assignments (weekly meetings, workshops, creating poster, abstract, presentation)

**Student Participant Assignments**

Pre-survey

Photo for abstract book

Elevator pitch (presented during one of the workshops or group meetings)

3 Evaluations

Final abstract for Abstract book

Research Paper

Poster

Powerpoint file of oral research presentation

Give oral presentation at Research Symposium

**Supplementary Material – Part 2 – Example of Faculty Mentor Report Form**

(modified from UIC SROP Handbook)

**Macromolecular Structure and Function Program** 2021

Faculty Mentor Report

Due: July 2th (#1), July 23 (#2), August 3 (#3)

Please submit the faculty mentor reports on the listed dates above. By the end of week 8, a total of 3 faculty mentor reports should be uploaded. Thank you for working with our scholars!

Faculty Mentor

Student

Week___ Average Weekly Contact Hours ____________

1) How well has the student met your expectations to date?

Low 1 2 3 4 5 High

Please explain

______________________________________________________________________

______________________________________________________________________

______________________________________________________________________

2) What preparations have you and the student been making on the symposium presentation and final research paper?

Please explain

______________________________________________________________________

______________________________________________________________________

______________________________________________________________________

______________________________________________________________________

3) Has the student completed all assignments in a timely manner?

Please explain

______________________________________________________________________

______________________________________________________________________

______________________________________________________________________

______________________________________________________________________

4) Has the student come to your meetings prepared to work on the research project?

Please explain

______________________________________________________________________

______________________________________________________________________

______________________________________________________________________

5) Please upload these forms to https://uofi.box.com/s/1nuhemvitftjyyaccael7ttepz7zfxno. If

you encounter difficulty uploading your report, please email them to Connie Jeffery at

cjeffery@uic.edu

Faculty mentor’s initials: ___

____________________________________________________________________

Faculty mentor’s signature: _______________________________________

Date: ________________________

E-mail address ______________________________

**Supplementary Material – Part 3 – Example of Student Participant Evaluation Form**

(modified from UIC SROP Handbook)

**Macromolecular Structure and Function Program 2021**

**Student Participant Evaluation Due: July 2th (#1), July 23 (#2), August 3 (#3)**

Please submit the report by the listed dates above. By the end of week 8, a total of 3 reports should be uploaded. Thank you!

**Due: __________________**

(To be completed by the student) Please fill out and upload to the MSFP Box site

**Please type the questions and answers*

1. Have you been able to maintain your timeline for research projects? How many pages of your draft have you completed so far and have you reviewed them with your mentor and writing consultants?
2. How is your presentation coming along? Have you begun putting your power point slides, transparencies, or poster board materials together? Have you reviewed your presentation with your mentor?
3. What other preparations if any will you need to complete in order to submit your final symposium presentation and research paper?

Name of student: _____________________________Date: _____________________

**Supplementary Material – Part 4 – Examples of Orientation/Tutorial Handouts**

**How to Keep a Lab Notebook**

(modified from http://www.colorado.edu/mcdb/MCDB3140/notebooks.html)

• Handwriting must be legible.
• All notes should be written in pen
• Errors should be crossed through with a single line, not erased or obliterated.
• Everything you do in the laboratory should be recorded in your lab notebooks, including notes, drawings, data, speculations, etc. Everything from your initial strategy through planning, execution and interpretation and should be in your notebook.
• Keep in mind that reports and presentations will be prepared from the notebook. You should have much more information recorded in your notebook than you can or should put on a poster or into a presentation.

• It’s important to note that an experiment must be thoroughly documented in your notebook if it is going to be presented in a talk, poster, paper, etc. If it is not described thoroughly and legibly in your notebook, it doesn’t count as having been done.

• Pages for each experiment should include:
• Title of experiment and date.
• The Hypothesis and/or Objective(s) of the experiment: what you are trying to do and why you are trying to do it.
• The Protocol for the experiment.
• Any deviations from your written procedure. This includes changes both intentional and accidental.
• Observations: everything that happens during your experiment that may have a bearing on the outcome or interpretation of the experiment (this includes color, precipitate, time, temperature, etc).
• Data: raw and calculated. Use complete sentences, tables and graphs where appropriate. Show sample calculations with steps and units.
• Discussion: Interpret your results. Refer back to your predictions. Draw conclusions about experiment. Make suggestions for further experiments or refinements to the procedure.

**Checklist for Students to Ensure Productivity**

(Adapted from the Council of Graduate Schools' Supervisory Practice Handbook)

• Have you tried to plan your work systematically?

• Have you identified the major difficulties?

• Do you understand the relevant references?

• Are your records in good order and could you answer a question on

something you did two weeks ago?

• Have you drafted the first version of any portion of the work that has been

completed?

• Do other people find your written work difficult to understand?

• Are there any tables, figures, or other matters that could usefully be prepared at

an early stage?

• Do those individuals in the department/laboratory you are working in

consider you a productive individual/member of the research team?

**Supplementary Material – Part 5 – Example of a Tutorial Worksheet**

**Tutorial on Databases and Servers**

**1. UniProt** database– <https://www.uniprot.org> - information about proteins, partly manually annotated and part from computer algorithms

search for ACE2 human, UniprotID [Q9BYF1](https://www.uniprot.org/uniprot/Q9BYF1)

additional names

functions

amino acid sequence in FASTA format

GOterms – Gene Ontology, standardized terminology

E.C. number

Cellular location

**2. BLAST** server– <https://blast.ncbi.nlm.nih.gov/Blast.cgi> - searching databases for similar DNA or protein sequences (and structures) and aligning them

Search with FASTA sequence from ACE2

What are top hits with “nonredundant” selected? What % identity? What % coverage?

What are top hits with model organisms? What % identity? What % coverage?

What are top nonhuman hits? What % identity? What % coverage?

Make multisequence alignments with different parameters

What are top PDB hits? What are the PDBIDs? What are % coverage? What are % identity? – fill in worksheet

**3. MoonProt** database–[www.moonlightingproteins.org](http://www.moonlightingproteins.org) – our lab’s database of proteins with 2 or more functions

what are other functions of ACE2?

**4. TMHMM** server - <http://www.cbs.dtu.dk/services/TMHMM/> - to look for TM helices, although this doesn’t find beta-barrel TM proteins

How many TM helices does ACE2 have?

Where are they located in the amino acid sequence?

How many TM helices does PGI have?

>sp|P06744|G6PI_HUMAN Glucose-6-phosphate isomerase OS=Homo sapiens OX=9606 GN=GPI PE=1 SV=4

MAALTRDPQFQKLQQWYREHRSELNLRRLFDANKDRFNHFSLTLNTNHGHILVDYSKNLV

TEDVMRMLVDLAKSRGVEAARERMFNGEKINYTEGRAVLHVALRNRSNTPILVDGKDVMP

EVNKVLDKMKSFCQRVRSGDWKGYTGKTITDVINIGIGGSDLGPLMVTEALKPYSSGGPR

VWYVSNIDGTHIAKTLAQLNPESSLFIIASKTFTTQETITNAETAKEWFLQAAKDPSAVA

KHFVALSTNTTKVKEFGIDPQNMFEFWDWVGGRYSLWSAIGLSIALHVGFDNFEQLLSGA

HWMDQHFRTTPLEKNAPVLLALLGIWYINCFGCETHAMLPYDQYLHRFAAYFQQGDMESN

GKYITKSGTRVDHQTGPIVWGEPGTNGQHAFYQLIHQGTKMIPCDFLIPVQTQHPIRKGL

HHKILLANFLAQTEALMRGKSTEEARKELQAAGKSPEDLERLLPHKVFEGNRPTNSIVFT

KLTPFMLGALVAMYEHKIFVQGIIWDINSFDQWGVELGKQLAKKIEPELDGSAQVTSHDA

STNGLINFIKQQREARVQ

How many TM helices does CXCR4 have?

>sp|P61073|CXCR4_HUMAN C-X-C chemokine receptor type 4 OS=Homo sapiens OX=9606 GN=CXCR4 PE=1 SV=1

MEGISIYTSDNYTEEMGSGDYDSMKEPCFREENANFNKIFLPTIYSIIFLTGIVGNGLVI

LVMGYQKKLRSMTDKYRLHLSVADLLFVITLPFWAVDAVANWYFGNFLCKAVHVIYTVNL

YSSVLILAFISLDRYLAIVHATNSQRPRKLLAEKVVYVGVWIPALLLTIPDFIFANVSEA

DDRYICDRFYPNDLWVVVFQFQHIMVGLILPGIVILSCYCIIISKLSHSKGHQKRKALKT

TVILILAFFACWLPYYIGISIDSFILLEIIKQGCEFENTVHKWISITEALAFFHCCLNPI

LYAFLGAKFKTSAQHALTSVSRGSSLKILSKGKRGGHSSVSTESESSSFHSS

How many TM helices does Tar have?

>sp|P07017|MCP2_ECOLI Methyl-accepting chemotaxis protein II OS=Escherichia coli (strain K12) OX=83333 GN=tar PE=1 SV=2

MINRIRVVTLLVMVLGVFALLQLISGSLFFSSLHHSQKSFVVSNQLREQQGELTSTWDLM

LQTRINLSRSAVRMMMDSSNQQSNAKVELLDSARKTLAQAATHYKKFKSMAPLPEMVATS

RNIDEKYKNYYTALTELIDYLDYGNTGAYFAQPTQGMQNAMGEAFAQYALSSEKLYRDIV

TDNADDYRFAQWQLAVIALVVVLILLVAWYGIRRMLLTPLAKIIAHIREIAGGNLANTLT

IDGRSEMGDLAQSVSHMQRSLTDTVTHVREGSDAIYAGTREIAAGNTDLSSRTEQQASAL

EETAASMEQLTATVKQNADNARQASQLAQSASDTAQHGGKVVDGVVKTMHEIADSSKKIA

DIISVIDGIAFQTNILALNAAVEAARAGEQGRGFAVVAGEVRNLASRSAQAAKEIKALIE

DSVSRVDTGSVLVESAGETMNNIVNAVTRVTDIMGEIASASDEQSRGIDQVALAVSEMDR

VTQQNASLVQESAAAAAALEEQASRLTQAVSAFRLAASPLTNKPQTPSRPASEQPPAQPR

LRIAEQDPNWETF
